# Supplementary material for: Bile Duct Ligation Upregulates Expression and Function of L-Amino Acid Transporter 1 at Blood–Brain Barrier of Rats via Activation of Aryl Hydrocarbon Receptor by Bilirubin
Source: Biomedicines. 2021 Sep 26;9(10):1320. doi: 10.3390/biomedicines9101320 (PMC8533316; doi:10.3390/biomedicines9101320)
Supplement: Supplementary file 1 [file biomedicines-09-01320-s001.zip › biomedicines-1332022-supplementary.pdf]

biomedicines

Article

Bile Duct Ligation Upregulates Expression and Function of L-Amino Acid Transporter 1 at Blood–Brain Barrier of Rats via Activation of Aryl Hydrocarbon Receptor by Bilirubin

Xiaoke Zheng †, Hanyu Yang †, Lan Qin, Siqian Wang, Lei Xie, Lu Yang, Weimin Kong, Liang Zhu, Li Liu * and Xiaodong Liu *

Center of Drug Metabolism and Pharmacokinetics, School of Pharmacy, China Pharmaceutical University, Nanjing 210009, China; zhengxk0923@yeah.net (X.Z.); shenyhycpu@163.com (H.Y.); 17878076997@163.com (L.Q.); siqianwang@yeah.net (S.W.); asdfghjkl951415@163.com (L.X.); yanglu2214@163.com (L.Y.); 1831010057@stu.cpu.edu.cn (W.K.); a8459022@126.com (L.Z.)

* Correspondence: liulee@cpu.edu.cn (L.L.); xdliu@cpu.edu.cn (X.L.); Tel.: +86-025-8327-1006 (X.L.)

†These authors equally contributed to this work.

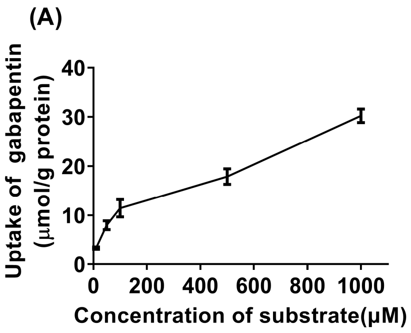

Figure S1. Effects of incubation time and concentration of gabapentin in uptake system on the cellular uptake of gabapentin. Data are presented as the mean ± SD ($n = 6$).

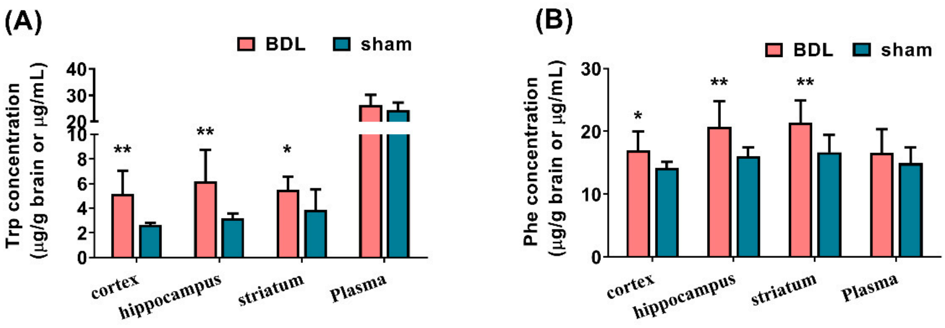

Figure S2. Effects of BDL-induced liver failure on Trp and Phe distributions in brain tissues. * $p < 0.05$ and ** $p < 0.01$ versus sham rats.

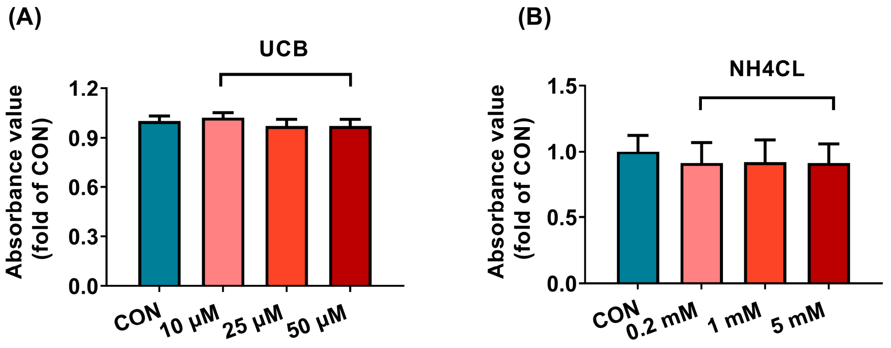

Figure S3. Effects of UCB and NH4Cl on cell viability, at the concentrations tested. Data are presented as the mean ± SD (n = 6).

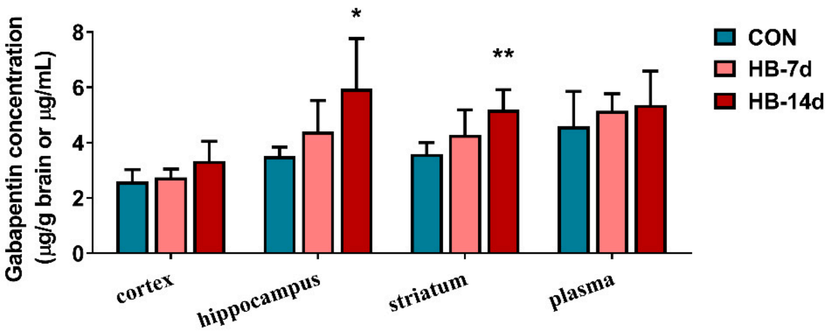

Figure S4. Concentrations of gabapentin in the cortex, hippocampus, striatum, and plasma of rats. Data are presented as the mean ± SD (n = 6). * $p < 0.05$ and ** $p < 0.01$ versus CON rats.

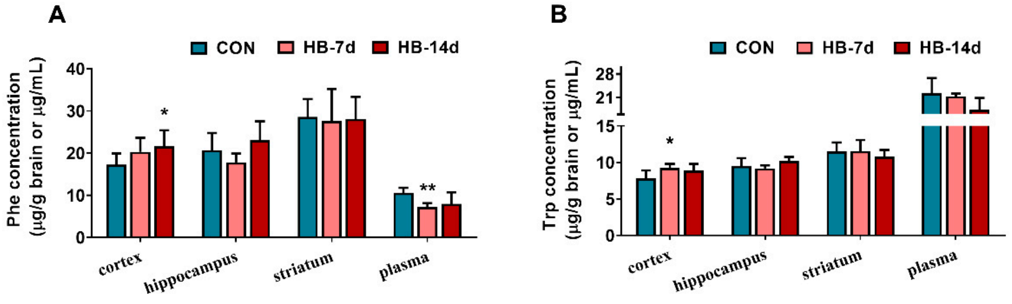

Figure S5. Effects of hyperbilirubinemia on Trp and Phe distributions in brain tissues of rats. Concentrations of Phe (**A**) and Trp (**B**) in cortex, hippocampus, striatum, and plasma of rats. Data are presented as the mean ± SD (n = 5). * $p < 0.05$ and ** $p < 0.01$ versus sham rats.
